# LAMP assay for the detection of the Asian citrus psyllid, *Diaphorina citri* Kuwayama (Hemiptera: Psylloidea: Psyllidae)

**DOI:** 10.1038/s41598-023-37721-w

**Published:** 2023-07-05

**Authors:** Arati Agarwal, Francesco Martoni, Lixin Eow, Brendan C. Rodoni, Mark J. Blacket

**Affiliations:** 1 Agriculture Victoria Research, AgriBio - Centre for AgriBioscience, 5 Ring Road, Bundoora, VIC 3083 Australia; 2 School of Applied Systems Biology, La Trobe University, Bundoora, VIC 3083 Australia

**Keywords:** Molecular biology, Entomology

## Abstract

*Diaphorina citri* Kuwayama, also known as the Asian citrus psyllid (ACP), can vector the bacterium *Candidatus* Liberibacter asiaticus (CLas), agent of Huanglongbing (HLB): an incurable disease affecting citrus trees worldwide. In citrus growing regions where ACP and HLB are absent, such as Australia, the risk of an incursion and consequent economic damage to citrus industries make this psyllid one of the top-priority pests. Due to ACP’s small dimensions and the generally poorly studied native psylloid fauna worldwide, morphological identification of this insect to distinguish it from harmless species is challenging, especially in the field, and with immature, partial or damaged specimens. To allow rapid and efficient detection of ACP in the field, we designed and optimised a new Loop-mediated isothermal amplification (LAMP) assay for the detection of *D. citri* based on the mitochondrial 16S locus. The optimised ACP 16S LAMP assay produced amplification from *D. citri* samples within 13.3 ± 3.6 min, with an anneal derivative of ~ 78.5 °C. A synthetic gBlock gene fragment was also developed to be used as positive control for the new LAMP assay with a different anneal derivative of ~ 83 °C. An existing commercially available LAMP assay for detection of the bacterium CLas was also tested in this study on ACP DNA. The ACP 16S LAMP assay we developed and tested here provides a valuable new in-field compatible tool that can allow early detections of ACP, enabling a quick biosecurity response, and could potentially be adopted by a wide range of users, from farmers to agronomists and from researchers to industry.

## Introduction

Psyllids, also known as jumping plant-lice, belong to the superfamily Psylloidea (Hemiptera: Sternorrhyncha), which comprises about 4000 described species worldwide across seven families^1^. These sap-feeding insects are generally highly specialised with respect to the plant species on which they develop and feed^2^, making some species important economic pests for agriculture and forestry both due to their feeding damage and to their ability to vector plant pathogens^3,4^. Amongst these, *Diaphorina citri* Kuwayama (Hemiptera: Psyllidae), the Asian citrus psyllid (ACP), is considered one of the most damaging citrus pests^5^ for its ability to transmit two *Candidatus* Liberibacter species in the field: *Ca.* L. asiaticus (CLas) and *Ca.* L. americanus (CLam)^6^. These Liberibacter species, together with *Ca*. L. africanus, transmitted by the African citrus psyllid *Trioza erytreae* Del Guercio (Triozidae), are the three bacterial agents which can cause Huanglongbing (HLB), regarded as the most economically devastating citrus disease worldwide^7^.

The severity of HLB as a citrus disease and the pathways with which ACP can be introduced in new areas, both due to weather events and human introductions^6^, make this psyllid a very important pest. Due to these aspects, the early phases of ACP detection in a new incursion are key to biosecurity responses aiming to eradicate or limit the risk of its establishment^8,9^. Morphological identification of this insect, especially in the field, is challenging due to ACP’s small size and to some of its diagnostic characters—such as the male parameres—requiring microscopic examination^10^. Additionally, the high number of undescribed and/or uncharacterised species of Psylloidea worldwide^1^, makes the process of distinguishing ACP from harmless species not only challenging, but often limited to operators with strong entomological taxonomic skills. Furthermore, no exhaustive key to the species of *Diaphorina* has been published, and identification is still done by comparison against older descriptions^10^. Within Australia, while the markings on the ACP wings are considered to be useful morphological characters, these are very similar to the markings on the wings of species of the genus *Acizzia* (Hemiptera: Psyllidae), a species-rich genus in Australia, where it comprises at least forty described species^2,11–13^, but is also present worldwide^1,2^.

Molecular assays may offer an alternative method for an efficient identification of ACP. For example, standard DNA barcoding targeting the subunit I of the *Cytochrome oxidase* gene (COI;^14^) has been successfully applied to psyllids^15–17^. However, from collection to sequencing, this process may take several days to achieve a diagnosis, highlighting a lack of rapid, in-field compatible molecular tests for detection of ACP.

LAMP, Loop-mediated isothermal amplification^18^, is a highly target-specific, rapid method for amplification of DNA which can be conducted in the laboratory or field in portable devices producing identification results in less than one hour. Several LAMP assays have recently been developed for diagnosing priority plant pests such as Queensland fruit fly *Bactrocera tryoni* (Froggatt)^19^, grape phylloxera *Daktulosphaira vitifoliae* (Fitch)^20^, Khapra beetle *Trogoderma granarium* Everts^21^, and fall armyworm *Spodoptera frugiperda* (J. E. Smith)^22^, proving this technique can be an extremely valuable tool for in-field detection of pests. Like qPCR^23^ LAMP assays can also employ gBlocks Gene Fragment (gBlock) as targeted synthetic oligonucleotides as controls to monitor assay performance^22^.

While there are at least four different LAMP assays which have been developed for the detection of CLas^24–27^, no LAMP assay has been developed for the detection of ACP, the potential insect vector, to date. Here we developed the first molecular diagnostic LAMP assay to detect the Asian citrus psyllid which has been fully optimised and tested for laboratory and in-field use.

In this study we: (1) developed a new ACP LAMP assay based on the mitochondrial 16S locus, (2) tested a broad panel of non-target psyllid species to assess the specificity of the new LAMP assay, (3) tested in-field non-destructive DNA extraction methods, (4) designed a gBlock to use as a synthetic positive control, as well as (5) validated a commercially available LAMP assay for CLas, which ACP can transmit.

## Results

### ACP 16S LAMP assay design and optimisation

**Figure 1:**
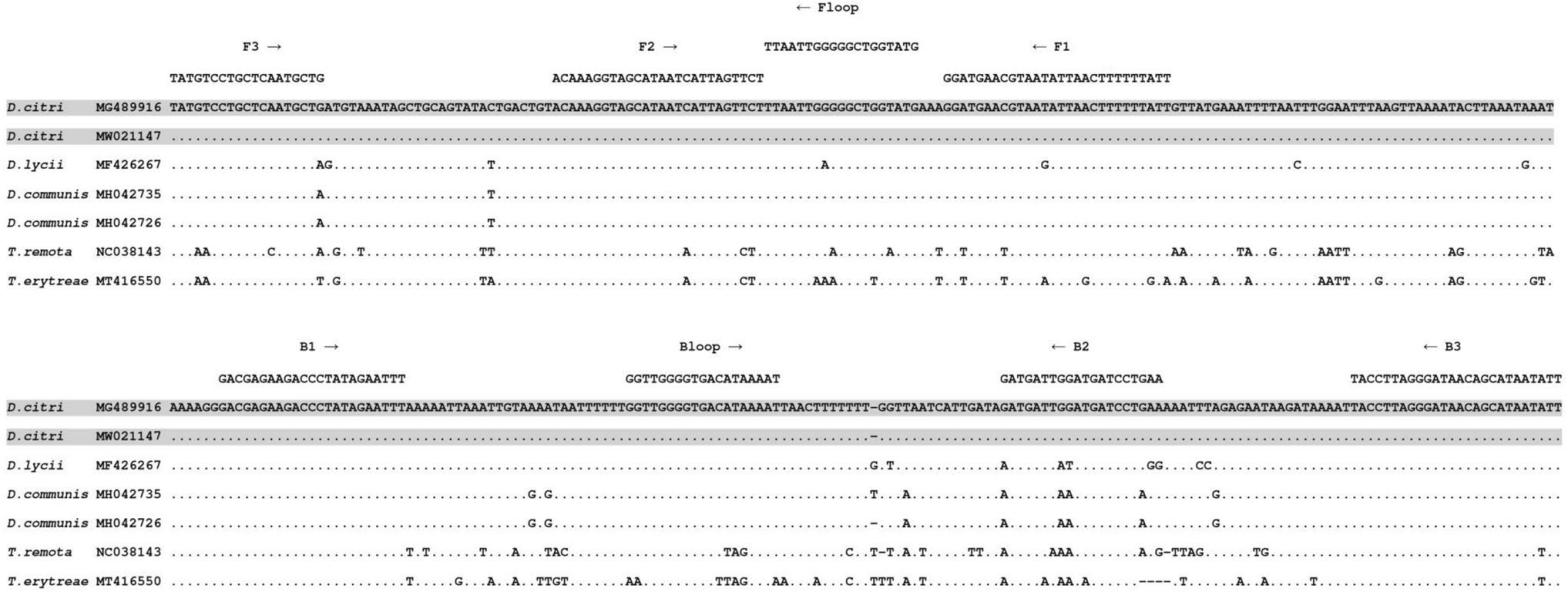
DNA sequence alignment of the mitochondrial 16S locus from *Diaphorina citri* (ACP) and other psyllids used for primer design for the ACP 16S LAMP assay. Grey shading highlights ACP. Primers are indicated above the alignment.

LAMP primers (Table 1, Supp Fig. 1) were developed to target a 340 bp portion of the 16S gene (Fig. 1). Six primers were designed in the ACP 16S LAMP assay, two inner primers (FIP and BIP) and two outer primers (F3 and B3). The addition of loop primers (Floop and Bloop) helped in generating a faster reaction hence reducing the time of positive amplification. The optimised primer ratio (F3/B3: FIP/BIP: Floop/Bloop) was determined to be 1:8:4, with final primer concentrations of 0.4 µM, 3.2 µM and 1.6 µM, respectively.

**Table 1:** Asian citrus psyllid (ACP) 16S LAMP primer and amplicon (gBlock) sequences and parameters.

Primer	Sequence 5′–3′	Primer Length (bp)	Predicted Tm, annealing temperature °C	Degeneracy of primer (fold)
ACP_16S_F3	TATGTCCTGCTCAATGCTG	19	50	None
ACP_16S_B3	AATATTATGCTGTTATCCCTAAGGTA	26	59	None
ACP_16S_Floop	CATACCAGCCCCCAATTAA	54	73.5	None
ACP_16S_Bloop	GGTTGGGGTGACATAAAAT	43	77.2	None
ACP_16S_FIP	AATAAAAAAGTTAATATTACGTTCATCC**ACAAAGGTAGCATAATCATTAGTTCT**	19	62.1	None
ACP_16S_BIP	GACGAGAAGACCCTATAGAATTT**TTCAGGATCATCCAATCATC**	19	58.6	None
gBlock Fragment	cccTATGTCCTGCTCAATGCTGcccACAAAGGTAGCATAATCATTAGTTCTcccTTAATTGGGGGCTGGTATGcccGGATGAACGTAATATTAACTTTTTTATTcccGACGAGAAGACCCTATAGAATTTcccGGTTGGGGTGACATAAAATcccGATGATTGGATGATCCTGAAcccTACCTTAGGGATAACAGCATAATATTccc	207	N/A	N/A

The F2 and B2 primer regions of the FIP and BIP primers are marked in bold and underlined. Lowercase letters in the gBlock indicate extra c’s added between LAMP primer sites to increase the overall Tm of the amplicon.

### Specimens examined

**Figure 2:**
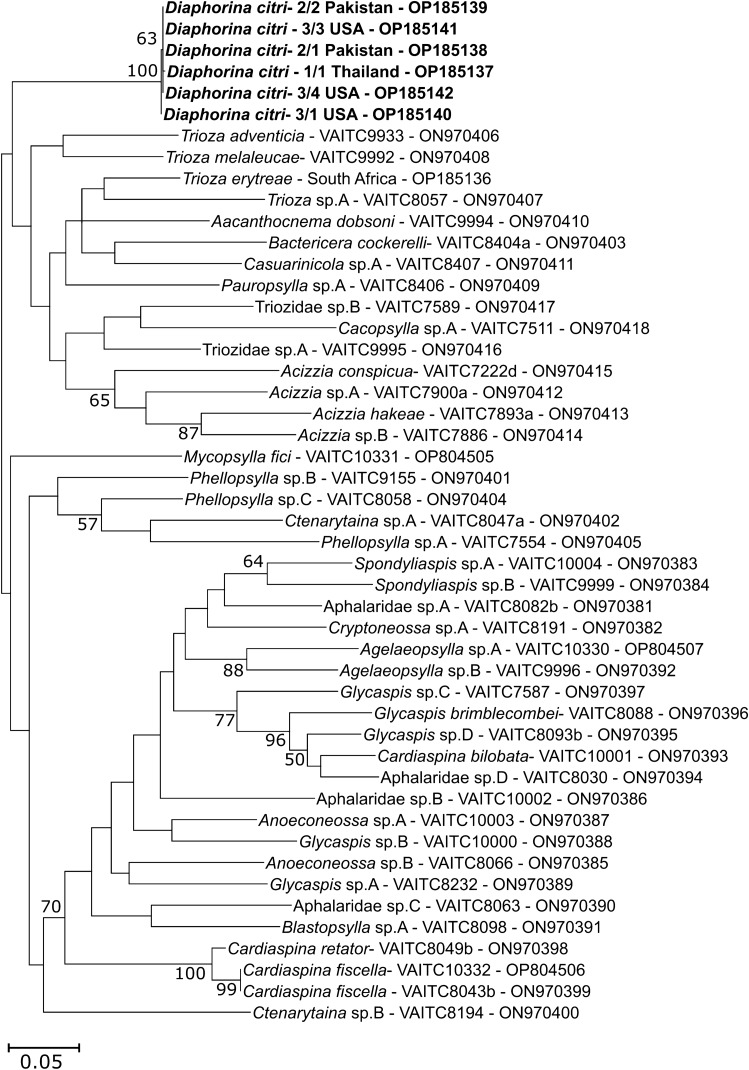
Maximum Likelihood tree (5,000 bootstrap replicates) developed to confirm species identifications and assess the genetic variation in the sequence of mitochondrial *cytochrome oxidase, subunit I* (COI) between ACP and other Australian native and exotic psyllids sequenced in this study. Bootstrap values < 50% are not reported. The target species, *D. citri*, is indicated in bold at the top of the tree, distinct from the other taxa. The scale bar is a genetic distance of 5%. All COI sequences were generated in this study, GenBank accession numbers are indicated together with the VAITC database identification number.

All specimens examined in the current study were initially identified morphologically, based on the latest taxonomical classification^1^, and confirmed through DNA barcoding (Table 2). COI sequences of 48 specimens were generated in this study and submitted to GenBank, with accession numbers ON970381–ON970418, OP185136–OP185142, and OP804505–OP804507 (Table 2). COI sequences from 41 different species (in addition to *D. citri*), represent four families within the Psylloidea (Aphalaridae, Carsidaridae, Psyllidae and Triozidae) (Fig. 2). Of these, at least 29 sequences (~ 70%) belong to species that are either undescribed or previously molecularly uncharacterised.

**Table 2:** Panel of psyllid specimens used for developing the ACP 16S LAMP assay.

Species	Family	ID	COI Acc. #	ACP 16S LAMP		
				Time (min)	Temp (^o^C)	+/−
Diaphorina citri *	**Psyllidae**	**1/1 F Thailand**	**OP185137**	**13.5**	**78.7**	** + **
Diaphorina citri *	**Psyllidae**	**2/1 Pakistan**	**OP185138**	**12.75**	**78.3**	** + **
Diaphorina citri *	**Psyllidae**	**2/2 F Pakistan**	**OP185139**	**11**	**78.4**	** + **
Diaphorina citri *	**Psyllidae**	**3/1M USA**	**OP185140**	**14.5**	**78.3**	** + **
Diaphorina citri *	**Psyllidae**	**3/3 USA**	**OP185141**	**10.5**	**78.3**	** + **
Diaphorina citri *	**Psyllidae**	**3/4 USA**	**OP185142**	**10**	**78.7**	** + **
*Agelaeopsylla* sp. A	Aphalaridae	VAITC 9996	ON970392			−
*Agelaeopsylla* sp. B **	Aphalaridae	VAITC 10330	OP804507			−
*Anoeconeossa* sp. A	Aphalaridae	VAITC 10003	ON970387			−
*Anoeconeossa* sp. B	Aphalaridae	VAITC 8066	ON970385			−
Aphalaridae sp. A	Aphalaridae	VAITC 8082b	ON970381			−
Aphalaridae sp. B	Aphalaridae	VAITC 10002	ON970386			−
Aphalaridae sp. C	Aphalaridae	VAITC 8063	ON970390			−
Aphalaridae sp. D	Aphalaridae	VAITC 8030	ON970394			−
*Blastopsylla* sp.	Aphalaridae	VAITC 8098	ON970391			−
*Cardiaspina bilobata*	Aphalaridae	VAITC 10,001	ON970393			−
*Cardiaspina fiscella*	Aphalaridae	VAITC 8043b	ON970399			−
*Cardiaspina fiscella* **	Aphalaridae	VAITC 10332	OP804506			−
*Cardiaspina retator*	Aphalaridae	VAITC 8049b	ON970398			−
*Cryptoneossa* sp.	Aphalaridae	VAITC 8191	ON970382			−
*Ctenarytaina* sp. A	Aphalaridae	VAITC 8047b	ON970402			−
*Ctenarytaina* sp. B	Aphalaridae	VAITC 8194	ON970400			−
*Glycaspis brimblecombei*	Aphalaridae	VAITC 8088	ON970396			−
*Glycaspis* sp. A	Aphalaridae	VAITC 8232	ON970389			−
*Glycaspis* sp. B	Aphalaridae	VAITC 10000	ON970388			−
*Glycaspis* sp. C	Aphalaridae	VAITC 7587	ON970397			−
*Glycaspis* sp. D	Aphalaridae	VAITC 8093b	ON970395			−
*Phellopsylla* sp. A	Aphalaridae	VAITC 7554	ON970405			−
*Phellopsylla* sp. B	Aphalaridae	VAITC 9155	ON970401			−
*Phellopsylla* sp. C	Aphalaridae	VAITC 8058	ON970404			−
*Spondyliaspis* sp. A	Aphalaridae	VAITC 10004	ON970383			−
*Spondyliaspis* sp. B	Aphalaridae	VAITC 9999	ON970384			−
Mycopsylla fici**	Carsidaridae	VAITC 10331	OP804505			−
*Acizzia conspicua*	Psyllidae	VAITC 7222d	ON970415			−
*Acizzia hakeae*	Psyllidae	VAITC 7893a	ON970413			−
*Acizzia* sp. A	Psyllidae	VAITC 7900a	ON970412			−
*Acizzia* sp. B	Psyllidae	VAITC 7886	ON970414			
*Cacopsylla* sp.	Psyllidae	VAITC 7511	ON970418			−
*Aacanthocnema dobsoni*	Triozidae	VAITC 9994	ON970410			−
Bactericera cockerelli *	Triozidae	VAITC 8404a	ON970403			−
*Casuarinicola* sp.	Triozidae	VAITC 8407	ON970411			−
*Pauropsylla* sp. A	Triozidae	VAITC 8406	ON970409			−
*Trioza adventicia*	Triozidae	VAITC 9933	ON970406			−
Trioza erytreae *	Triozidae	S. Africa	OP185136			−
*Trioza melaleucae*	Triozidae	VAITC 9992	ON970408			−
*Trioza* sp. A	Triozidae	VAITC 8057	ON970407			−
Triozidae sp. A	Triozidae	VAITC 9995	ON970416			−
Triozidae sp. B	Triozidae	VAITC 7589	ON970417			−

For each specimen, species and family are reported, as well as the identification, the accession number for the COI sequences generated in this study and the results of the ACP 16S LAMP assay. The COI sequences presented here are the same as used in Fig. 2, and have been used to provide DNA barcoding identification of psyllid species. Bold indicates the target species, *Diaphorina citri.*

*Exotic species.

**This species was tested in the field using the QuickExtract DNA extraction method.

### Evaluation of ACP gBlock Gene fragment

**Figure 3:**
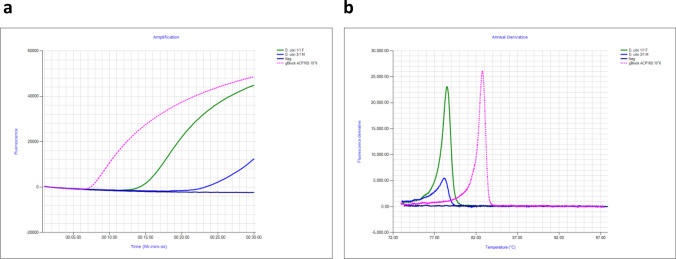
Amplification profile and anneal derivative curve comparison of ACP gBlock gene fragment (synthetic positive control), and samples of *D. citri*. (**a**) Amplification profile of *D. citri* samples at 16 and 22 min and gBlock 1 × 10^6^ copies/µL, at ~ 8 min (pink). Negative, no amplification (purple). (**b**) Anneal derivative of LAMP amplicons showing two peaks, ~ 78.5 °C for *D. citri* and ~ 83 °C for the gBlock (pink).

The detection sensitivity of the 207 bp ACP gBlock was found to be sensitive, amplifying as low as ~ 1 × 10^3^ copies/µL of ACP gBlock within 30 min with an anneal derivative of ~ 83 °C. One million copies (1 × 10^6^) of gBlock amplified in 8 min, which is earlier than the amplification time of two *D. citri* samples which amplified in 16 and 22 min (Fig. 3a). Based on this amplification time, 1 × 10^6^ copies/µL of ACP gBlock was found to be suitable for use as synthetic positive in ACP 16S LAMP assay. The anneal derivative of LAMP amplicons produced two distinct peaks (Fig. 3b), ~ 78.5 °C for *D. citri* DNA (see below) and ~ 83 °C for the gBlock. The negative no-template control did not amplify, neither did it produce an anneal peak.

### ACP 16S LAMP assay specificity results

**Figure 4:**
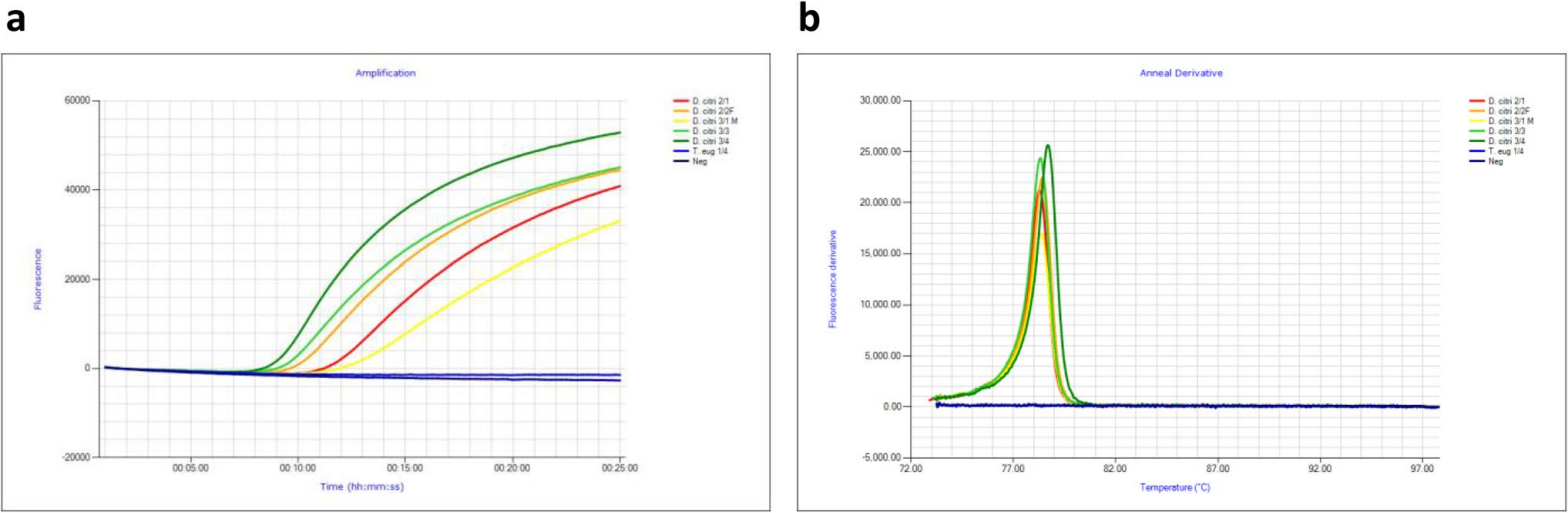
Optimised ACP 16S LAMP assay results using 1:8:4 primer ratio. (**a**) Positive amplification profile of 5 *D. citri* samples within 10 to 20 min and one non-target *Trioza* species no amplification (blue). Negative flat line (purple). (**b**) Anneal derivative at ~ 78.5 °C for 5 *D. citri* samples. Negative and *Trioza* species, no anneal peak (purple and blue).

All *D. citri* specimens (n = 6) tested (Table 2, Fig. 2) produced positive amplification for the ACP 16S LAMP assay (Fig. 4a), while none of the non-target psyllid samples (n = 42), belonging to at least 18 genera and four families, amplified DNA. *D. citri* DNA produced positive amplification in 13.3 ± 3.6 min, with amplification within 20 min considered as positive. All six *D. citri* samples produced an anneal profile at ~ 78.5 °C (Fig. 4b, Table 2). None of the negative non-template controls amplified in any of the LAMP runs tested for optimising this assay confirming the absence of primer dimer and reagent contamination (Fig. 4).

The non-destructive QuickExtract DNA extraction method was demonstrated in the field, using three non-target psyllid specimens of *Mycopsylla fici* (Carsidaridae), and a specimen each of *Agelaeopsylla* sp. and *Cardiaspina fiscella* (both Aphalaridae) (Table 2). None of the non-target samples and negative non-template control amplified, except for the gBlock 1 × 10^6^ copies/µL which amplified in 8.25 min with an anneal derivative temperature of 82.7 °C.

### *Candidatus* Liberibacter asiaticus LAMP assay results

**Figure 5:**
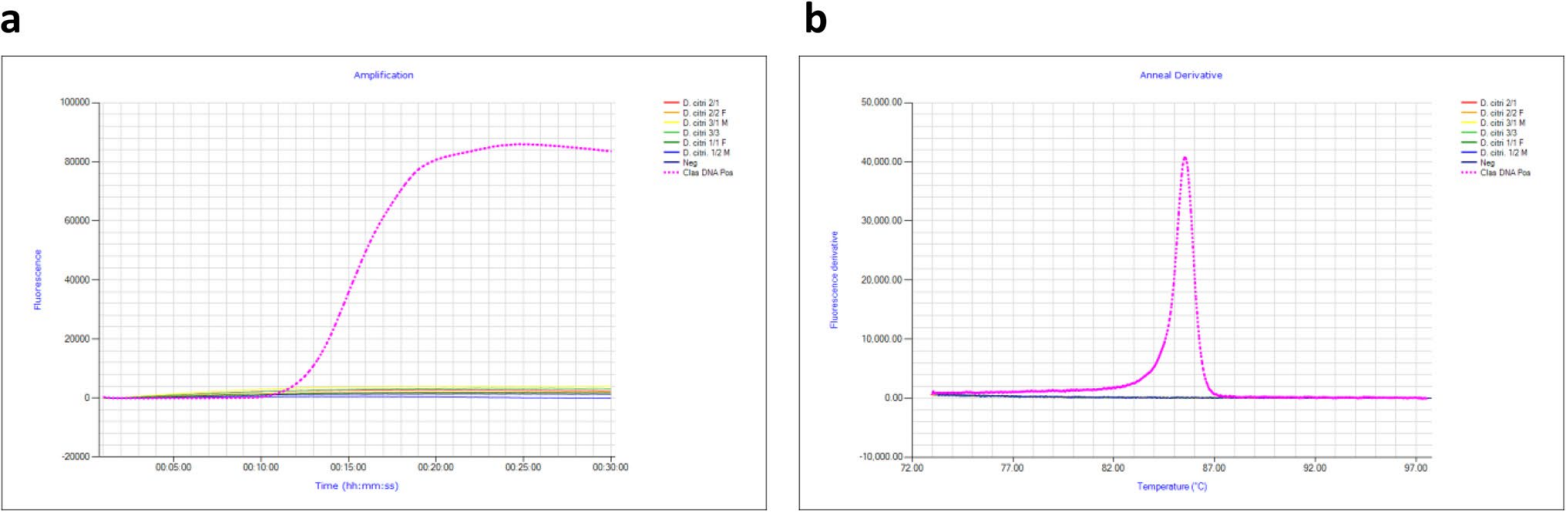
Amplification profile and anneal derivative of *Candidatus* Liberibacter asiaticus (CLas) LAMP assay. (**a**) Amplification profile of CLas DNA (synthetic positive) within 15 min (pink). (**b**) Anneal derivative at ~ 85.5 °C for CLas positive (pink). All the 6 *D. citri* samples tested were negative, no amplification (flat line). Negative, no amplification and no anneal peak (purple).

None of the *D. citri* DNA samples (n = 6) tested for the presence of the bacterium CLas produced positive amplification (Table 3, Fig. 5a). The recommended DNA template used in the kit for the LAMP assay was 5 µL of positive DNA which amplified in 9.75 min. We found that using a lower volume of 2 µL of positive DNA increased the amplification time by only approx. 5 min at 14.25 min. The anneal derivative temperature was 85.4 °C (Table 3, Fig. 5b). The negative non-template control did not amplify confirming that there was no contamination in the reagents and the primers performed as expected (Table 3, Fig. 5).

**Table 3:** DNA samples of psyllid species *Diaphorina citri* (ACP) and *Candidatus* Liberibacter asiaticus (CLas), tested for the CLas LAMP assay.

Sample #	Species	Template (µL)	PK-C.lib_asiaticus-050W		LAMP
			Time (min)	Temp (°C)	
1	*D. citri* 1/1 F	2	N/A	0.0	Negative
2	*D. citri* 2/1	2	N/A	0.0	Negative
3	*D. citri* 2/2 F	2	N/A	0.0	Negative
4	*D. citri* 3/1 M	2	N/A	0.0	Negative
5	*D. citri* 3/3	2	N/A	0.0	Negative
6	*D. citri* 3/4	2	N/A	0.0	Negative
	CLas DNA*	**5**	**9.75**	**85.3**	**Positive**
**7**	CLas DNA*	**2**	**14.25**	**85.5**	**Positive**
8	Neg (NTC)	1	N/A	0.0	Negative

For each sample species name, template and results of the CLas LAMP assay are reported. Bold indicates synthetic positive CLas DNA.

*Synthetic positive control included in the commercial kit.

## Discussion

Reliable identification of psyllids in the field is of paramount importance for early detection and management of pest species, such as ACP^9^. However, identification of these insects can be extremely challenging without taxonomic expertise, especially when dealing with immature stages; with many species being morphologically uncharacterised^1^ or appearing very similar to one another^1,2^. Likewise, adult specimens collected in the field often have incomplete morphology, being damaged by commonly used trapping methods, such as sticky traps. Furthermore, psyllids can easily be windblown to plants that are not their host,which can lead to psyllids collected from citrus plants being incorrectly suspected of being exotic pest species^29^. Molecular techniques for the identification of psyllids can be used as valuable tools for diagnostics and surveillance. DNA-based diagnostic techniques can be used by non-taxonomists, are generally less time-consuming than morphological examination, and can be applied to partial, immature or damaged specimens.

In designing and optimising this LAMP assay for ACP, we also generated and deposited on public database DNA sequences of psyllid taxa mostly native to Australia, that can now be used for diagnostics using the DNA barcoding technique^14^. Here, we generated 48 partial COI sequences across four families within the Psylloidea. These have proven to be a valuable tool to assess the genetic diversity of the psyllid species tested in this study, clearly separating the target species, ACP, from all others. Furthermore, these COI sequences provide additional genetic information on the diversity of the Australian Psylloidea. COI sequences from 41 different species were generated in this study (in addition to ACP), contributing to the number of works that have used COI as a marker for species delimitation and biodiversity assessments for this insect group^15–17^. Of these, 29 sequences (~ 70%) belong to species that are either undescribed or previously uncharacterised, therefore providing important genetic information for this poorly studied group^17^.

The main aim of this work, however, was to design and optimise a new LAMP assay for rapid detection of Asian citrus psyllid for both laboratory and in-field use. This test will be a useful tool in regions of the world where ACP is currently absent and diagnostic tests in the field are required within surveillance programs, but also in those areas where ACP is present, to monitor seasonal fluctuations in the populations and range expansions across different properties. While the ACP 16S LAMP assay we have developed has been optimised for use on a relatively expensive portable real-time fluorometer, it is likely that the assay will work on other technologically simpler LAMP platforms, such as colorimetric systems, as we have previously demonstrated for assays we have developed for other pests^21,22^.

In order to make this LAMP assay functional and ready-to-use specifically in those countries or regions where ACP is not present, such as Australia, we designed and optimised a synthetic DNA positive control (gBlock) for use in ACP 16S LAMP assays (following^20–22^). This synthetic DNA is beneficial in: (i) providing a consistent supply of positive control DNA as ACP is an exotic psyllid and often hard to get hold of positive samples for optimising the LAMP assay, (ii) providing a consistent control to allow tracking of the performance of LAMP assays across runs, (iii) providing a positive control that is easily differentiated from LAMP amplification of ACP insect DNA, possessing a different annealing temperature.

To further aid the use of this LAMP assay in the field, the QuickExtract non-destructive DNA extraction method can be performed using the Genie III machine. Considering the small size of ACP and different life stages of the insect, using 25 µL of QuickExtract buffer would yield higher DNA amount producing faster amplification time in the LAMP assay as shown in previous studies^20^.

In the current study, laboratory testing of the ACP 16S LAMP assay was performed only on DNA extracted from adult ACP specimens. This LAMP assay could not be tested on samples from other life stages, including eggs or immatures, as these were not available. Indeed, it is often difficult to source specimens of exotic species to use for testing new molecular assays (e.g.^28)^. However, LAMP assays developed for other insects have been shown to work on all life stages^19,20^. Given the sensitivity of our LAMP assay, i.e., down to ~ 1 × 10^3^ copies/µL of ACP gBlock, it is anticipated that this LAMP assay would be able to accurately identify these early life stages of ACP.

Our validation of a currently available CLas assay showed that this test performed well, and could be used with low amounts of template, demonstrating the potential sensitivity of this assay and potential cost savings associated with using lower quantities of commercially available positive control DNA. The new ACP 16S LAMP assay we developed here together with the commercial CLas LAMP test now provide complementary tools for rapid laboratory or in-field detection and management of this potentially devastating citrus pest and pathogen, using highly specific rapid molecular LAMP assays.

## Materials and methods

### Psyllid samples and DNA extractions

DNA extracts of *D. citri* (n = 6) were sourced from the Victorian Agricultural Insect Tissue Collection (VAITC) and used for the development and testing of the new LAMP assay. Psyllid species (n = 41) belonging to at least 18 genera (Table 2), mostly native to Australia, as well as some key native and exotic pest species, such as *Trioza melaleucae*^29^, *Trioza erytreae* and *Bactericera cockerelli*, were sourced from the Victorian Agricultural Insect Collection (VAIC). These were used to assess the genetic distances between ACP and other psyllids tested, and to test the LAMP assay’s specificity. These samples were chosen to include a diverse set of species and genera, representative of the Australian psyllid fauna. DNA extractions of these psyllids were performed using DNeasy Blood and Tissue kit (Qiagen, Germany), as previously described^30^ for individual insects, using a non-destructive approach (except VAITC7222d, destructive approach).

Non-destructive DNA extraction is a pre-requisite for conducting LAMP assay in the field. The LAMP assay is more sensitive and less impacted by impurities present in the DNA extract, compared to real-time PCR, and can amplify from crude DNA extracts efficiently. DNA of additional individual psyllids (n = 3), from an additional two species, was extracted *in loco* (Table 2), from single adult psyllids using QuickExtract DNA extraction solution 1.0 (Epicentre, USA) following the protocol presented elsewhere^19^.

### Marker selection, isolation and amplification

#### DNA barcode COI reference sequences

Based on numerous studies commonly utilising the COI barcode region for psyllid identification (e.g.^15–17^), and the known suitability of this marker for LAMP assays (e.g.^19,20^, DNA sequences of the COI locus were obtained from ACP and other non-target psyllids. PCRs were conducted using the primer pair PsyCOI-F3 (5′-ACAATTGTTACWGCWCAYGC-3′;^17^) and HCO2198 (5′-TAAACTTCAGGGTGACCAAAAAATCA-3′;^31^). PCR cycling conditions started with a denaturation phase at 94 °C for 2 min followed by 40 cycles of denaturation at 94 °C for 30 s, annealing at 50 °C for 45 s and extension at 72 °C for 45 s. A final extension phase was carried out for 2 min at 72 °C. PCR amplificons were run on agarose gels (1%) and sequenced commercially by Macrogen Inc. (Macrogen, Seoul, Korea) in both directions. Consensus sequences were manually assembled by combining and trimming forward and reverse sequences, and then aligned using MEGA X^32^. The Kimura-2-parameter model (K2P^33^), a Maximum likelihood (ML) algorithm with a bootstrap of 5,000 replicates was used to generate a COI gene tree to visualise the genetic distance between the psyllids analysed here.

#### Development of a 16S LAMP assay for ACP

While the COI sequences (above) were utilised to estimate genetic relatedness and are extremely useful as barcoding marker for psyllids, this region appeared to be unsuitable for development of a LAMP assay. Our initial attempt to develop a COI LAMP assay using this region (data not shown) proved unsuccessful due to the presence of extensive primer dimer interactions between LAMP primers.

A second attempt to design the ACP LAMP assay was made using an alternative mitochondrial locus, the 16S gene, based on DNA sequences obtained from GenBank, outlined in^34^ (Supp Fig. S1). Two ACP individuals, representing different 16S haplotypes, and the closest genetic relative *Diaphorina communis* were included in a DNA alignment, along with the most similar reference DNA sequences from GenBank (Fig. 1). LAMP primers (Table 1) were developed to target a 340 bp region of the ACP 16S locus, which appears to be conserved within ACP, but shows a small number of differences from other psyllids (Fig. 1). Six new LAMP primers were manually designed by eye to target eight DNA regions from the 16S reference alignment (Fig. 1). The complete set of LAMP primers (Table 1) was analysed together to detect potential primer-dimer interactions using the Thermo Fisher Multiple Primer Analyzer tool (www. thermofisher.com). Primers were synthesized by Sigma (Australia).

### ACP 16S LAMP assay optimisation

The primer ratio (F3/B3: FIP/BIP: Floop/Bloop) for this assay was tested and optimised following a published protocol^19^. The final optimised primer master mix was prepared by adding the specified amount of each of the six primers in a 1:8:4 ratio (see below). A 100 μL volume of primer master mix 1:8:4 (F3/B3: FIP/BIP: Floop/Bloop) was prepared by adding 10 μL (10 μM) each of F3/B3, 8 μL (100 μM) each of FIP/BIP, 4 μL (100 μM) each of Floop/Bloop and 56 μL of Ultrapure water (Invitrogen, Australia).

Each LAMP reaction mix was made by adding 10 µL of primer master mix to 14 µL of Isothermal Master Mix (ISO-004, OptiGene, UK) and 1 µL of template DNA into each well of the Genie strip (25 µL total reaction volume). Each run included a positive control (i.e., 1 × 10^6^ gBlock), a no-template negative control, and six test samples. All LAMP assays were run in the Genie III at 65 °C for 25 min followed by an annealing curve analysis from 98 °C to 73 °C with ramping at 0.05 °C/s. The total run time being approximately 35 min. The run files were transferred and analysed using a PC version of the software Genie Explorer version 2.0.7.11.

### Evaluation of a gBlock Gene Fragment for ACP 16S LAMP assay

A gBlock dsDNA fragment (Integrated DNA Technologies, Iowa, USA) was designed for use as synthetic DNA positive control for the ACP 16S LAMP assay. This synthetic fragment consisted solely of concatenated LAMP primers separated by runs of “ccc”, to increase the overall Tm of the gBlock (Table 1). To evaluate detection sensitivity, the copy number and a ten-fold serial dilution (1:10) of the gBlock was prepared as outlined in^20^. Sensitivity of the LAMP assay was tested using the serially diluted (1 × 10^8^ to 1 × 10 copies/µL) of gBlock in the Genie III, following the same ACP 16S LAMP assay conditions as mentioned above, with the run time increased from 25 to 30 min, to allow detection of the gBlock present at low concentrations. Following this another LAMP run was conducted using two *D. citri* DNA samples to compare amplification time with a standard amount of one million copies (1 × 10^6^ copies/µL) of gBlock.

### Test for commercial LAMP assay kit for the *Candidatus* Liberibacter asiaticus (CLas) optimisation

A Commercially available CLas LAMP assay kit (Cat. No. PK-C.lib_asiaticus050W) was sourced for testing in the current study following the manufacturer’s recommendations (OptiGene, UK). Each LAMP reaction mix was made by adding 5 µL of primer master mix to 15 µL of Isothermal Master Mix (ISO-004, OptiGene, UK) and 2 µL of template DNA (recommended 5 µL of DNA) into each well of the Genie strip. Both 5 µL and 2 µL of template DNA were tested in two separate Genie runs. Each run included a CLas synthetic positive control (i.e., Cat. No. CD-CLas_050), a no-template negative control, and six *D. citri* DNA test samples. All LAMP assays were run in the Genie III at 65 °C for 30 min followed by an annealing curve analysis from 98 °C to 73 °C with ramping at 0.05 °C/s and results analysed on the blue channel.

## Supplementary Information

Supplementary Figure 1.

## Data Availability

GenBank, accession numbers ON970381-ON970418, OP185136-OP185142, and OP804505-OP804507.
