# Supplementary material for: LAMP assay for the detection of the Asian citrus psyllid, Diaphorina citri Kuwayama (Hemiptera: Psylloidea: Psyllidae)
Source: Sci Rep. 2023 Jul 5;13:10895. doi: 10.1038/s41598-023-37721-w (PMC10322826; doi:10.1038/s41598-023-37721-w)

Supplementary Figure 1. Maximum Likelihood tree based on DNA sequences of the 16S locus. Species include: *Diaphorina citri* (ACP), two closely related *Diaphorina* species, and two *Trioza* species used as outgroups. *D.=Diaphorina*, *T.=Trioza.* GenBank accession numbers are shown after species names. Numbers on nodes indicate bootstrap values. (Modified from [34]).

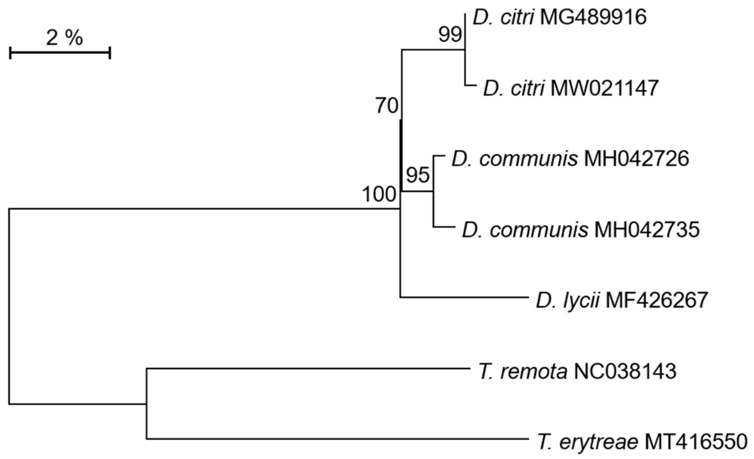

Supplement: Supplementary file 1 — Supplementary Figure 1. [file 41598_2023_37721_MOESM1_ESM.pdf]
